# The Effects of Subchronic Exposure to Terbuthylazine on Early Developmental Stages of Common Carp

**DOI:** 10.1100/2012/615920

**Published:** 2012-05-02

**Authors:** Stanislava Štěpánová, Lucie Plhalová, Petra Doleželová, Miroslav Prokeš, Petr Maršálek, Miša Škorič, Zdeňka Svobodová

**Affiliations:** ^1^Department of Veterinary Public Health and Toxicology, Faculty of Veterinary Hygiene and Ecology, University of Veterinary and Pharmaceutical Sciences, Palackého 1/3, 612 42 Brno, Czech Republic; ^2^Institute of Vertebrate Biology, Academy of Sciences of the Czech Republic, v.v.i., Květná 8, 603 65 Brno, Czech Republic; ^3^Department of Pathological Morphology and Parasitology, Faculty of Veterinary Medicine, University of Veterinary and Pharmaceutical Sciences, Palackého 1/3, 612 42 Brno, Czech Republic

## Abstract

The aim of this study was to assess the impact of terbuthylazine in surface waters on fish under experimental conditions. Subchronic toxic effects on embryos and larvae of common carp (*Cyprinus carpio*) were investigated during a 30-day toxicity test. The exposure to terbuthylazin showed no effect on mortality, but significant differences (*P* < 0.0001) were revealed on weight and growth parameters at concentrations of 520 and 820 *μ*g/L. The inhibition of specific growth rate at concentrations of 520 and 820 *μ*g/L was 14% compared to the control group. No significant negative effects on total body length and body weight were observed at lower concentrations (0.9 and 160 *μ*g/L). The concentrations 520 and 820 *μ*g/L were associated with a delay in development compared to other experimental groups and controls. On the basis of weight and growth rate evaluation and determination of developmental stages, the No Observed Effect Concentration (NOEC) of terbuthylazine was estimated at 160 *μ*g/L and the Lowest Observed Effect Concentration (LOEC) was 520 *μ*g/L. According to these results, the reported environmental concentration of terbuthylazine in Czech rivers does not impact growth, development, morphology, or histology of carp embryos and larvae.

## 1. Introduction

Terbuthylazinee (N2-tert-butyl-6-chloro-N4-ethyl-1,3,5-triazine-2,4-diamine), a triazine herbicide, is a selective systemic herbicide which acts as a photosynthesis inhibitor. It is used as a broad spectrum herbicide in maize, sorghum, vines, citrus, coffee, potatoes, legumes, and forestry [1]. It is taken up through roots and leaves and is distributed throughout the plant, which enables it to be used in both pre- and postemergence treatment. Terbuthylazine is used as a substitute for atrazine, which has been banned in many countries.

Terbuthylazine is stable in neutral, weakly acid, and weakly alkaline media. Degradation of terbuthylazine in aquatic environment depends on the presence of sediments and biological activity. The half-life in water at 20°C is 8 days at pH 1, 86 days at pH 5, >200 days at pH 9, and 12 days at pH 13. In sunlight the half-life is >40 days. The major metabolic process in animals is N-dealkylation and oxidation [1]. Its solubility in water is 8.5 mg/L at 20°C, and log Kow is 3.04 [2].

Triazine pesticides are divided into two groups: asymmetrical triazines or triazinones, such as metribuzin, and symmetrical triazines. The symmetrical triazines (*s*-triazines) can be further divided into chloro-*s*-triazines (e.g., atrazine, terbuthylazine, simazine, propazin, and cyanazine), thiomethyl-*s*-triazines (ametryn, prometryn, terbutryn), and methoxy-*s*-triazine (e.g., prometron) [3].

Triazine herbicides are for animals relatively nontoxic during acute exposure and well tolerated during long-term administration. They are not considered a developmental or reproductive toxin, are not mutagenic or carcinogenic, and are not irritating to skin or eyes [3]. On the contrary, some studies of the effects of atrazine report alterations in reproduction, the endocrine system, and development or increased incidence of mammary tumors in rats [4].

The extensive use of pesticides in agriculture results in environmental contamination. In Czech Republic it was according to the Czech State Phytosanitary Administration applied about 107 000 kg of terbuthylazine in 2010. The Czech Hydrometeorological Institute has reported atrazine, cyanazine, metamitron, metribuzin, prometryn, simazine, terbuthylazine, and terbutryn in Czech rivers. Terbuthylazine was present in about 50% of water samples taken in the years 2005 to 2009. The highest environmental concentrations reached 0.1 *μ*g/L in 2005 to 2.8 *μ*g/L in 2006 (2.6 *μ*g/L in 2009) [5]. Monitoring of terbuthylazine in groundwater, surface water, and drinking water has been undertaken by many authors [6–10]. Terbuthylazine levels were monitored in the River Po (Italy) from 1988 to 1991 and concentrations ranging from less than detection limits up to 0.3 *μ*g/L were reported [6]. Terbuthylazine contamination of the aquifer in Italy was monitored from 1998 to 2004, concentrations never exceeded the EU drinking water limit (0.1 *μ*g/L); the maximum concentration was 0.04 *μ*g/L. Concentrations ranging from less than detection limits up to 1.27 *μ*g/L were reported in surface and groundwater in northern Spain by Hildebrandt et al. [8]. Terbuthylazine concentrations in Spain in the range of 0.02–0.39 *μ*g/L were reported by Castillo et al. [7]. The presence of terbuthylazine in drinking water in Athens was less than detection limits up to 0.02 *μ*g/L [10].

Studies of the toxicity of various triazine herbicides to fish indicate that it can cause growth retardation and morphological, biochemical, hematological, histopathological, and physiological alteration [11–20], but less is known about the specific effects of terbuthylazine on fish.

The aim of this study was to assess effects of subchronic exposure to sublethal concentrations of terbuthylazine on growth, development, and histology of embryolarval developmental stages of common carp (*Cyprinus carpio*).

## 2. Material and Methods

Testing solutions were prepared from Click 500 SC. The active compound is terbuthylazine at a concentration of 500 g/L. The tested concentrations were prepared by using the stock solution (100 mg/L of terbuthylazine), which included the solvent dimethylsulfoxide (DMSO) to improve solubility. The highest tested concentration of terbuthylazine included 0.005 mL/L DMSO.

### 2.1. Experimental Protocol

Embryolarval toxicity tests were carried out using a modified protocol according to the OECD guideline 210 (fish, early-life stage toxicity test) [21]. Fertilized eggs of common carp were obtained from commercial fish farm. Eggs were produced according to standard methods of artificial reproduction as described by Kocour et al. [22].

Twenty-four hours after fertilization, 100 fertilized eggs were separated from unfertilized eggs and randomly distributed into fifteen crystallization dishes containing one of four ascending concentrations of terbuthylazine solution, or to a control dish (terbuthylazine-free tap water). The experiment was conducted in triplicate (a total of 300 fertilized eggs for each concentration and control). Tested concentrations were selected according to the environmental concentration, with the lowest tested concentration corresponding to the environmental concentration: 0.9, 160, 520, and 820 *μ*g/L. Concentrations during the test did not sink below 80% of the nominal concentration.

A semistatic method was used in which the testing solution was replaced twice daily. Hatching and survival were also observed twice daily, and dead embryos and larvae were removed. During the test, larvae were fed freshly hatched *Artemia salina ad libitum* twice a day prior to the bath exchange. The temperature, pH, and oxygen saturation were recorded daily.

The beginning of the test was designated day 1 (one day after fertilization). Hatching began on day 4 and was completed by day 6. Feeding with *A. salina* was initiated on day 7. The test was concluded on day 30, when all of the larvae in the control groups reached the juvenile stage.

During the test embryos and larvae were sampled to record developmental stage, length, weight, Fulton's condition factor (FCF), length-weight relationship, and morphological anomalies. Samples from each concentration and from the control were collected on day 6 (before feeding began) and on day 13 (15 fish, 5 from each replicate group), on day 20 (30 fish, 10 from each replicate group), on day 27 (15, 5 from each replicate group), and at the completion of the test on day 30 (45, 15 from each replicate group). Fish were fixed in 4% formalin.

### 2.2. Water Parameters

The basic physical and chemical parameters of tap water used in the tests were acid neutralization capacity (ANC_4.5_) 1.0–1.2 mmol/L; chemical oxygen demand (COD_Mn_) 0.8–1.2 mg/L; total ammonia below the limit of determination (<0.04 mg/L); NO_3_^−^ 11.2–13.5 mg/L; NO_2_^−^ below the limit of determination (<0.02 mg/L); Cl^−^ 10.2–12.5 mg/L; Σ Ca + Mg 3.01 mmol/L. The water temperature ranged from 19 to 22°C; pH was between 7.5 and 8.5, and dissolved oxygen did not fall below 60%.

### 2.3. Determination of Developmental Stages

The developmental stages were determined according to Penaz et al. [23] who have described nine embryonic (E1–E9), six larval (L1–L6), and two juvenile (J1-J2) stages in common carp. Total length (TL) was measured stereomicroscopically using a micrometer to 0.01 mm; weight (*W*) was measured to 0.1 mg.

### 2.4. Weight and Growth Rate Evaluation

Fulton's condition factor (FCF) was calculated at each sampling time:

(1) $$\text{FCF}=\frac{W\cdot 10^{5}}{L^{3}},$$

where *W* is the weight in g and *L* is the total length in mm.

The mean specific growth rate (SGR) was calculated for each experimental group beginning on day 6 (the first sampling time) and on day 30 (completion of the test):

(2) $$\text{SGR}=\frac{\bar{\ln w_{2}}-\bar{\ln w_{1}}}{t_{2}-t_{1}}\cdot 100,$$

(SGR: specific growth rate, *w*_1_: weight of one fish at time *t*_1_, *w*_2_: weight of one fish at time *t*_2_, *t*_1_: first sampling time, *t*_2_: end of the test).

The inhibition of specific growth (I) rate for each experimental group was calculated as follows:

(3) $$I\left[\%\right]=\frac{\text{SGR}\left(\text{control}\right)-\text{SGR}\left(\text{group}\right)}{\text{SGR}\left(\text{control}\right)}\cdot 100.$$

### 2.5. Statistical Analysis

Results were analyzed using STATISTICA 8.0 for Windows (StatSoft CR). After testing for normality of all parameters (Kolmogorov-Smirnov test) and homogenity of variances across groups (Levene's test), data were subjected to the parametric ANOVA Tukey's HSD test [24].

### 2.6. Histopathological Examination

The fish were prepared for histopathological examination, fixed in buffered 10% neutral formalin, dehydrated, embedded in paraffin wax, sectioned (cross-section) on a microtome at 4 *μ*m, and stained with hematoxylin and eosin (H&E). Histology of skin, gill, and liver was examined by light microscopy.

### 2.7. Measurement of Terbuthylazine

Gas chromatography with ion trap mass spectrometry (GC/IT-MS) was used for measurement of terbuthylazine. Sample preparation was based on simple liquid-liquid extraction into hexane.

Separation, identification, and quantification of terbuthylazine were based on the GC/IT-MS method. The gas chromatograph Varian 450-GC and VF-5 ms (30 m × 0.25 mm) column were used for separation. A Varian 220-MS ion trap mass spectrometer was used for identification and quantification. Chromatographic and MS conditions were based on methods described by Perreau and Einhorn [25]. All solvents were GC/MS-grade purity. Certified standard terbuthylazine was used.

Detection limit (3*σ*) of terbuthylazine is 0.01 *μ*g/L. Expanded uncertainty was 6.0% conditional on a coefficient of expansion of *k* = 2.

## 3. Results

### 3.1. Hatching

Eggs began hatching on day 4. The majority of the eggs hatched by day 5, and hatching was completed by day 6. No negative effects of terbuthylazine on hatching and viability of embryos at the tested concentrations were found.

### 3.2. Cumulative Mortality

No significant differences were found in total cumulative mortality among groups. In all experimental groups and control mortality was below 10%.

### 3.3. Length and Weight Parameters

**Figure 1:**
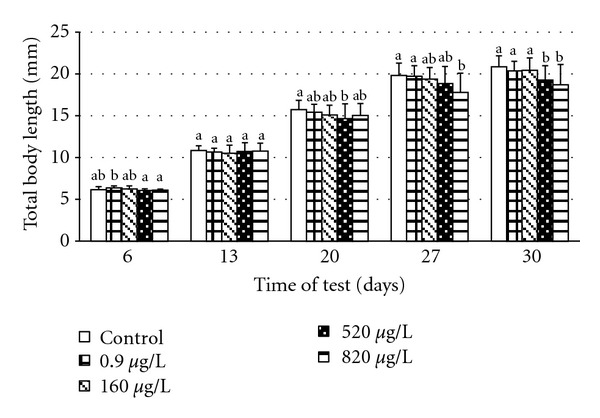
Total body length of common carp during the embryolarval toxicity test (mean ± SD; (a, b) groups with different alphabetic superscripts differ significantly (*P* < 0.05); day 6, 13, and 27 *n* = 75; day 20 *n* = 150; day 30 *n* = 225).

**Figure 2:**
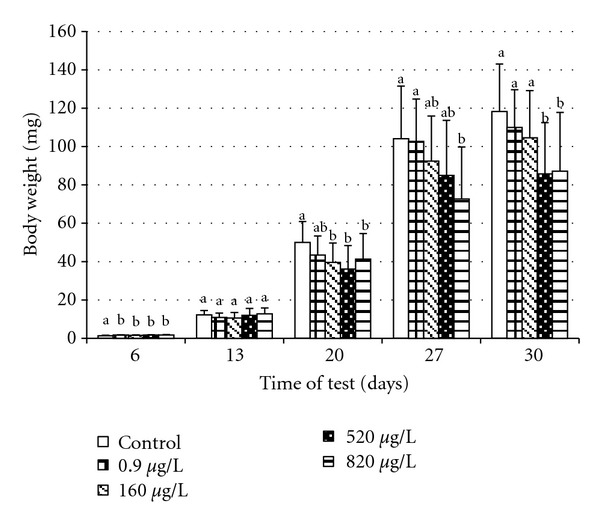
Body weight of common carp during the embryolarval toxicity test (mean ± SD; (a, b) groups with different alphabetic superscripts differ significantly (*P* < 0.05); day 6, 13, and 27 *n* = 75; day 20 *n* = 150; day 30 *n* = 225).

**Figure 3:**
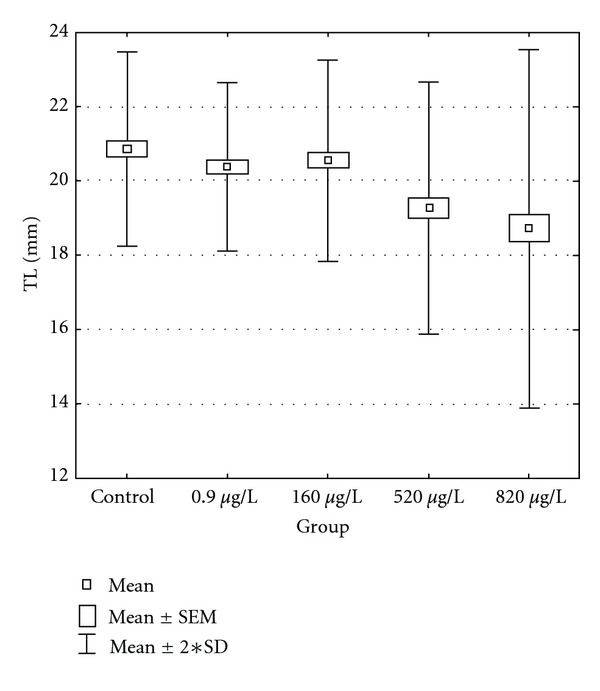
The variability of total body length of common carp after a 30-day embryolarval toxicity test; *n* = 225.

The time course for total body length (mm) and weight increase of the embryos and larvae in relation to terbuthylazine concentration in water are depicted in Figures 1 and 2. The influence of terbuthylazine concentration on body weight and body length of carp larvae began to be evident from day 20. Larval growth correlated with the tested concentrations. At the completion of the test, significant differences (*P* < 0.0001) in total length and weight were seen between control and experimental groups at 520 and 820 *μ*g/L. Total body length of fish in all groups at completion of the test is depicted in Figure 3. Greater variability was found in total body length between each fish in the same group in experimental groups 520 and 820 *μ*g/L compared with the control group.

The influence of terbuthylazine on FCF was not significant. The mean FCF value at the end of the test was 1.28 in control and 1.29, 1.20, 1.69, and 1.26 in tested concentrations 0.9, 160, 520, and 820 *μ*g/L, respectively.

Specific growth rate and inhibition of growth are shown in Table 1. The inhibition of specific growth rate was greatest at concentrations of 520 and 820 *μ*g/L; it was 14% compared to the control group.

### 3.4. Early Ontogeny

**Figure 4:**
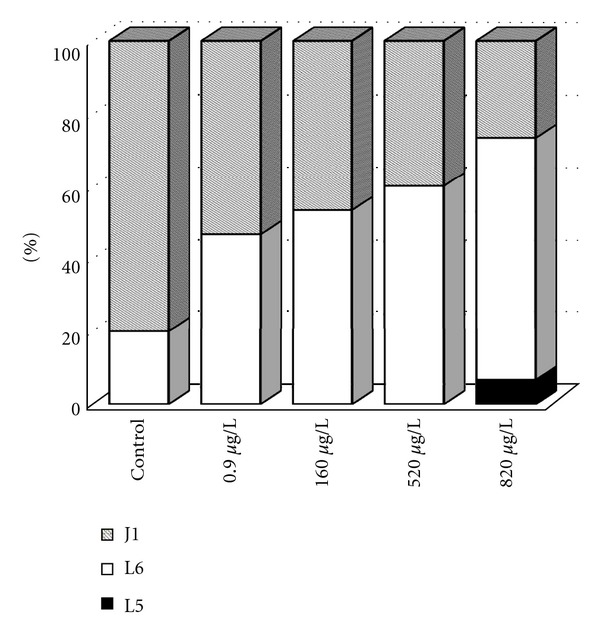
Developmental stages of common carp at day 27 (L5, L6-larval, and J1-juvenile developmental stages); *n* = 75.

Developmental stages were recorded at each sampling time in all groups. The differences in early ontogeny among control and experimental groups were evident at the completion of the test. Fish from the 520 and 820 *μ*g/L groups were significantly delayed in development (*P* < 0.0001) in a dose-dependent manner compared with the control group. After 30 days all fish in the control group reached the juvenile stage (J1). At terbuthylazine concentrations of 0.9 and 160 *μ*g/L, the rate was 96%, but at 520 and 820 *μ*g/L only 64% and 50%, respectively. The delay of development of carp at day 27 of the test is depicted in Figure 4, at day 27 reached 80% of fish juvenile stage (J1) in the control whereas only about 25% at 820 *μ*g/L.

### 3.5. Morphological Anomalies

No significant morphological anomalies were found. On day 13 elevated pigmentation in all fish exposed to 820 *μ*g/L was seen. At subsequent sampling times, this pattern was observed in individual larvae of the 820 *μ*g/L group. Pigmentation in other experimental groups was comparable with controls.

### 3.6. Histopathology

**Figure 5:**
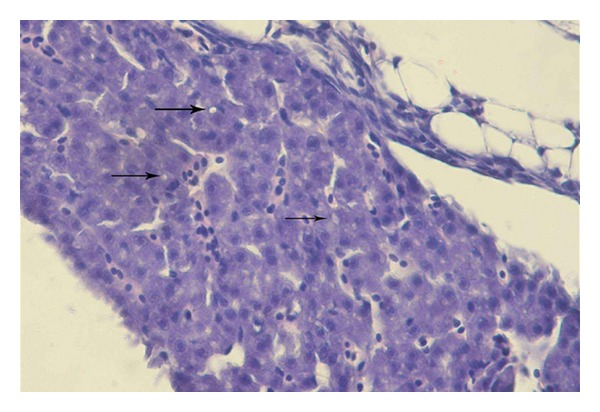
Vacuoles in the cytoplasm of hepatocytes of common carp exposed to the concentration of 820 *μ*g/L terbuthylazine for 30 days.

**Figure 6:**
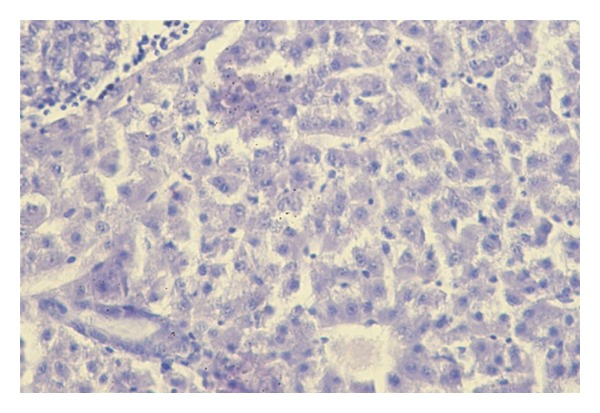
Section of liver of common carp from the control group.

Histological examination revealed lesions only in liver in fish exposed to 820 *μ*g/L after 30-days of the test. In liver were observed mild lesions including diffuse formation of small round to oval vacuoles in the cytoplasm of hepatocytes (Figure 5). Liver tissue was histologically comparable to that of the control (Figure 6). Tissue and organs of fish in experimental groups exposed to terbuthylazine at the concentrations of 0.9 *μ*g/L, 160 *μ*g/L, and 520 *μ*g/L exhibited no histological changes.

Based on growth parameters, developmental, and histological examination of all replications, No Observed Effect Concentration (NOEC) = 160 *μ*g/L and Lowest Observed Effect Concentration (LOEC) = 520 *μ*g/L were generated.

## 4. Discussion

Pesticides are serious pollutants of the aquatic environment that can have harmful effects on biota, including fish. Fish are subjected to prolonged exposure of small doses of contaminants in water that do not cause death but which could induce retardation of growth and developmental or histological changes. During the 30 day exposure of embryolarval developmental stages of carp to low concentrations of terbuthylazine (0.9, 160, 520, and 820 *μ*g/L), no effects on mortality of the exposed group compared to controls were found. The effect of terbuthylazine exposure was observable on length and weight growth. Retardation of growth in the experimental group compared to control became evident on day 20. After 30 days of exposure significantly smaller fish were found at exposure to 520 and 820 *μ*g/L. Variability in fish growth is influenced by a range of biotic and abiotic factors. The most important abiotic factors are temperature, light, oxygen concentration, and pH, along with water contamination and other factors [26]. All biotic and abiotic factors were identical except for the different concentration of the toxic substance. It is possible that the increasing individual variability in fish growth is influenced by the presence of terbuthylazine.

Some studies have indicated that fish mortality is the most sensitive parameter upon exposure to the *s*-triazine herbicides [11, 27]. According to our results of embryolarval test on carp, growth seems to be a more sensitive parameter than mortality. Arufe et al. [11] in a study of the toxic effect of a terbutryn-triasulfuron mixture to seabream (*Sparus aurata* L.) larvae found no terbutryn concentration that affected weight that did not also cause 100% mortality. Similar results were reported by Neskovic et al. [14] who did not find any significant change in carp body weight after exposure to atrazine. Growth reduction of *Galaxias maculates* after exposure to low concentrations of atrazine was observed by Davies et al. [28]. Plhalova et al. [15] reported growth reduction of *Danio rerio* after 28 days of exposure to terbutryn. In other studies, low doses of triazine herbicides on fish that did not induce mortality or retard growth but affected hematological and biochemical profile of common carp and rainbow trout have been reported [13, 16–18].

Histological examination revealed lesions in the livers of carp after 30 days of exposure to 820 *μ*g/L of terbuthylazine. The liver is the primary organ of metabolism and excretion of pollutants. It is able to detoxify toxic compounds, but their elevated concentrations can result in structural damage. Dezfuli et al. [29] reported severe damage to liver and gill of *Dicentrarchus labrax* after exposure to terbuthylazine. This included myelin-like structures and acute cell swelling of hepatocytes in the liver and necrosis, lamellar and cellular edema, epithelial lifting, telangiectasia, and fusion of secondary lamellae in the gills. Histological alterations in the liver of seabream (*Sparus aurata* L.) after exposure to a terbutryn-triasulfuron mixture were reported by Arufe et al. [11]. These alterations included nuclear pyknosis and cellular alterations related to shape of hepatocytes caused by the amount of lipid inclusions. Biagianti-Risbourg and Bastide [12] found a number of abnormalities in liver of juvenile grey mullet (*Liza ramada*) associated with exposure to atrazine. Changes in the liver after triazine exposure were also reported by Neskovic et al. [14] and Velisek et al. [17, 18]. Other reported histological lesions caused by triazines include changes to the respiratory epithelium and to the renal tubules of the caudal kidney [14–18].

Arufe et al. [11] identified morphological changes (curvature of the vertebral column) in some individuals after exposure to a terbutryn-triasulfuron mixture. Terbuthylazine did not induce morphological anomalies during the present trial, but delay in early development of carp was evident. After 30 days of exposure all fish in the control group reached the juvenile stage. At terbuthylazine concentrations of 0.9 and 160 *μ*g/L, the rate was 96%, but at 520 and 820 *μ*g/L only 64% and 50%, respectively. Disturbance of normal development and retardation in organogenesis of *D. rerio* embryos after atrazine exposure was observed by Wiegand et al. [20].

The advantage of toxicity tests on early-life stages of fish is the opportunity to observe developmental and morphological changes during exposure. Embryolarval toxicity testing on fish is a sensitive method, since it covers two developmental stages which differ in susceptibility as a result of physiological and biochemical differences, with fish exposed to chemicals in critical periods of development including hatching, start of exogenous nutrition, and changes of respiratory function [23, 30].

As terbuthylazine is relatively insoluble, we used the solvent DMSO. In the highest tested concentration (820 *μ*g/L), the concentration of DMSO was 0.005 mL/L. Machova et al. [31] stated that the concentration of 1 mL/L of DMSO did not result in lethal effects, abnormalities, or changes in growth parameters during an embryolarval toxicity test on carp.

The subchronic toxic effect of terbuthylazine on embryolarval developmental stages expressed as the LOEC value was 160 *μ*g/L, and the NOEC value was 520 *μ*g/L. According to these results, the environmental concentration of terbuthylazine in Czech rivers should have no effect on growth, developmental, morphological, or histology of embryos and larval stages of carp.

## Figures and Tables

**Table 1:** Growth rate results of the 30-day embryolarval test on common carp. *w*_6_, *w*_30_ = mean fish weight in selected group after 6 and 30 days of exposure, SGR: mean specific growth rate after 24 days of exposure, I: inhibition of specific growth after 24 days of exposure.

Terbuthylazine (*μ*g/L)	0 (control)	0.9	160	520	820
*w*_6_ (mg)	1.31 ± 0.32	1.67 ± 0.15	1.61 ± 0.19	1.67 ± 0.26	1.69 ± 0.29
*w*_30_ (mg)	118.31 ± 24.72	110.03 ± 19.63	104.62 ± 24.55	85.81 ± 26.75	87.11 ± 30.76
SGR	18.8	17.4	17.3	16.2	16.1
I (%)	—	7.5	8.0	13.9	14.2
